# Single berry reconstitution prior to RNA-sequencing reveals novel insights into transcriptomic remodeling by leafroll virus infections in grapevines

**DOI:** 10.1038/s41598-020-69779-1

**Published:** 2020-07-31

**Authors:** Sana Ghaffari, Jean Sébastien Reynard, Markus Rienth

**Affiliations:** 1 CHANGINS-Changins, HES-SO University of Applied Sciences and Arts Western Switzerland, College for Viticulture and Enology, Nyon, Switzerland; 2 Higher Institute of Applied Biology of Medenine, Medenine, Tunisia; 3 Virology-Phytoplasmology Laboratory, Agroscope, Nyon, Switzerland

**Keywords:** Plant stress responses, Biotic, Plant sciences, Plant molecular biology

## Abstract

Leafroll viruses are among the most devastating pathogens in viticulture and are responsible for major economic losses in the wine industry. However, the molecular interactions underlying the effects on fruit quality deterioration are not well understood. The few molecular studies conducted on berries from infected vines, associated quality decreases with the repression of key genes in sugar transport and anthocyanin biosynthesis. Sampling protocols in these studies did however not account for berry heterogeneity and potential virus induced phenological shifts, which could have biased the molecular information. In the present study, we adopted an innovative individual berry sampling protocol to produce homogeneous batches for RNA extraction, thereby circumventing berry heterogeneity and compensating for virus induced phenological shifts. This way a characterization of the transcriptomic modulation by viral infections was possible and explain why our results differ significantly from previously reported repression of anthocyanin biosynthesis and sugar metabolism. The present study provides new insights into the berry transcriptome modulation by leafroll infection, highlighting the virus induced upregulation of plant innate immunity as well as an increased responsiveness of the early ripening berry to biotic stressors. The study furthermore emphasizes the importance of sampling protocols in physiological studies on grapevine berry metabolism.

## Introduction

Grapevines (*Vitis* spp.) are one of the most widely grown perennial fruit crops worldwide. With a total surface area of 7.4 million hectares, grapevine commercialization as fresh or dry fruit, wine and liquor is of considerable socioeconomic importance for many countries^1^.

Grapevine leafroll disease (GLD) is one of the most widespread and economically important virus associated diseases affecting grapevines, accounting for approximately 10–70% of the losses in grape production^2^. GLD occurs wherever grapevines are grown and causes delayed ripening of fruits, reduced yield, altered fruit pigmentation and decreased sugar concentration^3^. Currently, five identified viral species from the *Closteroviridae* family are associated with GLD and are termed grapevine leafroll associated virus (GLRaV). GLRaV-1, 3 and 4 are *ampeloviruses*, GLRaV-2 is a *closterovirus*, and GLRaV-7 is a member of the genus *Velarivirus*^4,5^. The most widespread GLRaVs worldwide are GLRaV-1 and GLRaV-3^6^. Although it is generally accepted that leafroll viruses are phloem limited, Kurth et al.^7^ showed that this is not entirely the case in the grapevine berry where it can spread via the mesocarp. It is thus likely that single berries have a different viral load depending on their sink strength, and developmental stage. Though, several scientific studies have characterized the effects of GLD on vine performance and berry quality^3,8^, the molecular events underlying the detrimental effects on fruit quality and development have been poorly understood and described to date^9^.

The few molecular studies on berries of GLD infected vines have correlated decreased sugar and total anthocyanin content with the repression of genes in related pathways^9^. Sampling in previous studies did not account for the intra-cluster berry heterogeneity of ripening grapes^10^ and the phenological shifts caused by GLD, which can significantly change the outcome of molecular studies and mask direct virus related transcriptomic effects^11–13^. This can be explained by heterogeneous berry ripening which, on a cluster scale, lasts between 40–60 days^14^ but is mainly due to high intra-cluster heterogeneity, since on a single berry level it lasts only 15 days^13^. Commonly applied sampling protocols involve mixing berries from whole clusters at different time points and thus various individual ripening stages of single berries. Such a strategy may however mask biotic or abiotic stress modulated gene expression.

Moreover, other previous molecular studies characterizing grapevine response to viral infection^15^ have been conducted under uncontrolled field conditions (different locations, pedoclimates, clonal materials or rootstocks), thus potentially resulting in significant experimental noise in gene expression.

Grapevine berry development is a complex process displaying a double sigmoidal pattern comprising two distinct growth phases separated by a lag phase^16^. Sugar and anthocyanin accumulation as well as malic acid degradation occur only during the ripening after green growth^17^. Here, we used what is, to our knowledge, the first strictly selective individual sampling strategy in a biotic stress study to characterize the molecular effects of berry metabolism from GLD infected vines. The use of samples from vines grown in the same experimental block and therefore under similar pedoclimatic conditions, provides a detailed picture of GLD-mediated transcriptomic reprograming of grapevine berries.

## Results

### Vine and berry physiological and biochemical analysis and batch reconstitution

Virus screening by ELISA and RT-qPCR (for GLRaV-1 and GLRaV-3) of all individual vines for all other viruses regulated in the Swiss certification scheme, as detailed in material and methods, were negative for all plants. GLRaV-1 was positive in all individuals of treatment 1 (T1) and for GLRaV-1 plus GLRaV-3 of treatment 2 (T2) and confirmed thus, that the inoculation with the two different viruses was successful.

Measurement of gas exchange showed a decrease in photosynthesis, which was not significant between uninfected controls (C) and GLRaV-1 infected (T1) vines, however reduced by coinfection with both viruses GLRaV-1&3 (T2; Supplementary Fig. S1A). This can be explained by a strong decline in F_m_ (maximal possible value for fluorescence) (F_m_) leading to a reduction in quantum yield (F_v_/F_m_) in T2 (Supplementary Fig. S1C). Bertamini et al.^18^, observed a similar reduction of F_v_/F_m_ explained by a substantial photoinhibition or down-regulation of PSII (Photosystem 2) by GLD.

Yield was not different between C and T1 but almost reduced to zero in T2 due to a very low fertility (clusters per vine). N-tester readings were significantly lower in infected vines (Supplementary Fig. S1B).

To circumvent the introduction of biases in gene expression data due to berry heterogeneity, and to account for phenological shifts caused by viral infection, we sampled single berries, froze them in N_2_ and ground them before individual HPLC analysis.

**Figure 1:**
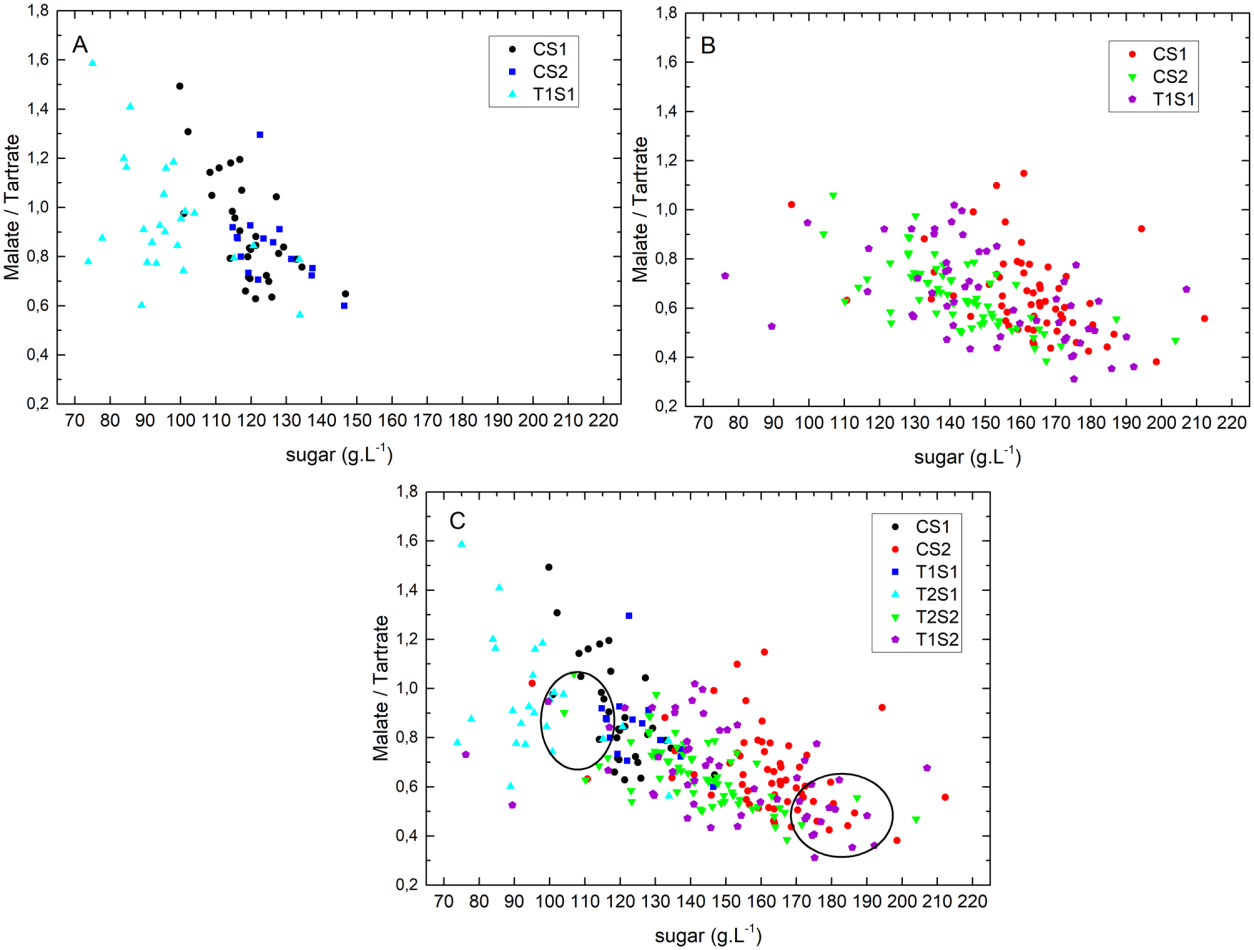
Malic acid/tartaric acid ratio as a function of sugar concentration from all sampled individual single berries from different treatments at stage 1 (**A**), stage 2 (**B**) and both stages together (**C**). Each point represents one berry. The circles in (**C**) contain the berries chosen for RNA extraction and subsequent RNA-seq.

Figure 1 illustrates the sugar concentration plotted against the malic to tartaric acid ratio (MA/TA) of all individually analyzed berries at stage 1 (Fig. 1A), stage 2 (Fig. 1B) and both stages together (Fig. 1C).

As expected, owing to the respiration of MA during ripening, the MA/TA ratio decreases throughout ripening when sugar concentration increases^17^. The biochemical analysis confirmed the rapid evolution of berries between the two temporarily close stages S1 (14.08.2017) and S2 (29.08.2017). A more than two-fold variation in sugar concentration is observed in the controls (C), ranging from 95 to 212 g L^−1^ (Fig. 1C) over a very short period of 15 days, confirming the very high asynchrony of individual berries previously reported in several studies^10,13^. This heterogeneity becomes even more pronounced in the berries of virus infected vines, which had a sugar concentration ranging from 89 to 207 g L^−1^ in T1 (GLRaV-1) vines and from 73 to 203 g L^−1^ in T2 (GLRaV-1&3) vines (Fig. 1C).

The distribution of all individually analysed berries (C, T1 and T2) sampled at S1 (Fig. 1A), shows that most T2 berries had a lower sugar concentration and higher MA/TA ratio than berries from non-infected vines (C), thus indicating a delay of phenology mainly caused by T2 in S1. Interestingly, this delay in phenology was less pronounced in S2, in which T1 and T2 berries were just slightly offset from C berries (Fig. 1B). In fact, T2 showed a berry sugar concentration 20% lower at S1 and 13% lower at S2 than those in controls. T1 showed a lower sugar concentration in berries only at S2 (6% lower than that in C; Table 1). Thus, we hypothesize that either T2 had the greatest effect on berry physiology during S1, during which it delayed berry ripening, or possibly the transition from the lag phase to véraison. Several abiotic factors, such as heat, are known to influence the onset of véraison^11,19^. T2 in S1 also appeared to have limited berry heterogeneity during early ripening, probably because of a delay in the onset of véraison. It furthermore seems that to some extent a certain uncoupling of malic acid degradation and sugar accumulation is triggered by virus infection mainly apparent in S1 by T2. A similar observation has been made in previous studies, where cool temperature delayed malic acid respiration from sugar accumulation potentially due to a better energetic state of plants under cool conditions^13^. A slightly delayed malic acid respiration in the presentation study could be explained by the fact that leaf virus inhibited sugar transport in the phloem or more likely due to an important change in the source-sink ratio of infected vines caused by lower yields (Supplementary Fig. S1C).

**Table 1:** Biochemical analysis of all sampled berries (“all”) and different berry batches selected for RNA-seq analysis (“selec”). (values are averages of at least four pooled berries with similar acid and sugar values).

Treatment + stage	Berry weight (g)	Sugar (g L^−1^)	MA (g L^−1^)	TA (g L^−1^)	MA/TA
CS1_all_	1.67 ± 0.36	119.16 ± 9.89	0.80 ± 0.23	0.89 ± 0.14	0.91 ± 0.21
CS1_selec_	1.74 ± 0.36	115.26 ± 1.19	0.78 ± 0.06	0.86 ± 0.05	0.91 ± 0.08
CS2_all_	1.70 ± 0.43	162.26 ± 18.03	0.56 ± 0.14	0.87 ± 0.14	0.65 ± 0.17
CS2_selec_	1.51 ± 0.27	172.33 ± 7.82	0.42 ± 0.07	0.85 ± 0.10	0.50 ± 0.05
T1S1_all_	1.63 ± 0.35	125.24 ± 9.32	0.87 ± 0.22	1.04 ± 0.24	0.84 ± 0.16
T1S1_selec_	1.79 ± 0.22	116.06 ± 0.95	1.03 ± 0.15	1.20 ± 0.23	0.87 ± 0.05
T1S2_all_	1.47 ± 0.28	152.03 ± 24.41	0.53 ± 0.16	0.80 ± 0.13	0.67 ± 0.19
T1S2_selec_	1.36 ± 0.35	177.71 ± 6.47	0.40 ± 0.05	0.84 ± 0.11	0.48 ± 0.05
T2S1_all_	1.26 ± 0.28	96.88 ± 15.48	0.80 ± 0.26	0.84 ± 0.20	0.95 ± 0.24
T2S1_selec_	1.33 ± 0.30	101.05 ± 1.79	0.73 ± 0.23	0.80 ± 0.18	0.90 ± 0.10
T2S2_all_	1.43 ± 0.27	142.51 ± 17.44	0.53 ± 0.14	0.81 ± 0.14	0.65 ± 0.13
T2S2_selec_	1.21 ± 0.12	165.94 ± 2.92	0.34 ± 0.03	0.73 ± 0.10	0.47 ± 0.06

*MA* Malate, *TA* tartrate.

± Standard deviation.

The high heterogeneity in Fig. 1 emphasizes the developmental differences or the degree of decoupling among individual berries. Batches of individual berries were reconstituted according to their biochemical characteristics (circles in Fig. 1C) before RNA extraction to obtain a phenology independent transcriptomic portrait of virus induced changes (Table 1).

### Global transcriptome analysis and expressed genes responding to GLRaV-1 and GLRaV-1&3 infections

RNA-seq analysis performed on reconstituted berry batches yielded a total of 360 million sequenced reads from all 18 samples.

**Figure 2:**
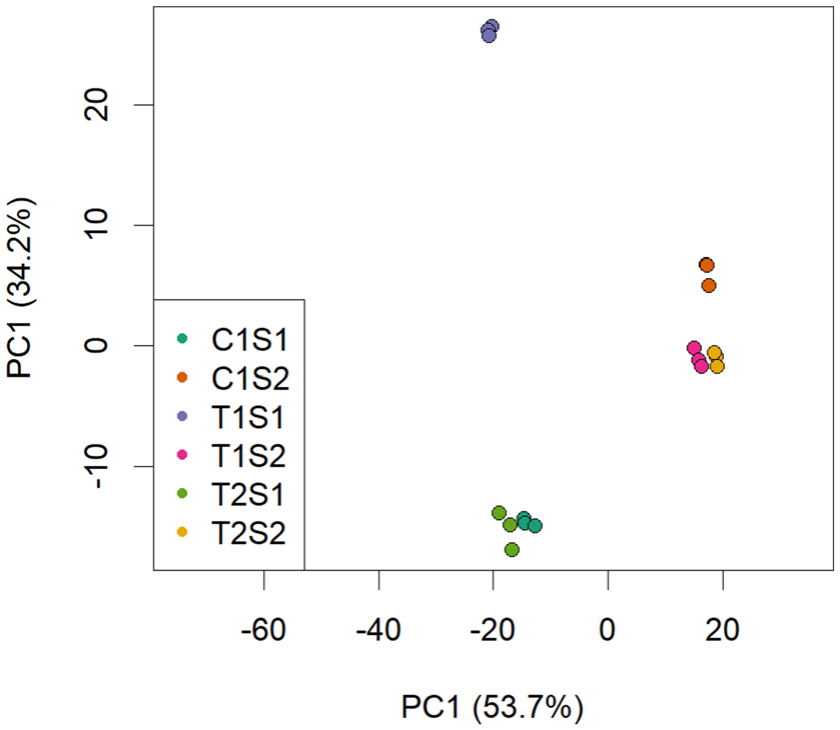
Principal component analysis of the whole normalized gene expression dataset for all treatments and stages, in triplicate.

Principal component analysis (PCA) on normalized gene expression (Fig. 2) confirmed the low variability among triplicates and showed that the expression data were consistent and reproducible between replicates and therefore reliable for further analysis.

A high portion of the overall variance in gene expression (88%) was explained by the first two principal components. PC1, accounting for 54% of the variation, separated the samples mainly by ripening stage, a result that may be explained by the transient berry transcriptome reprogramming between early and late ripening (between S1 and S2)^20,21^.

PC2 accounts for 34% of the variation and separates infected and control berries only at S1, indicating that viral infection (T1 and T2) had globally a smaller effect on the berry transcriptome at later ripening stage (S2).

Differential gene expression analysis yielded in a total of 2,136 differentially expressed genes (DEGs) (fold change > 2, pval adj < 0.05; see Supplementary Table S1) between the control and treatment groups in at least one of the two stages.

**Figure 3:**
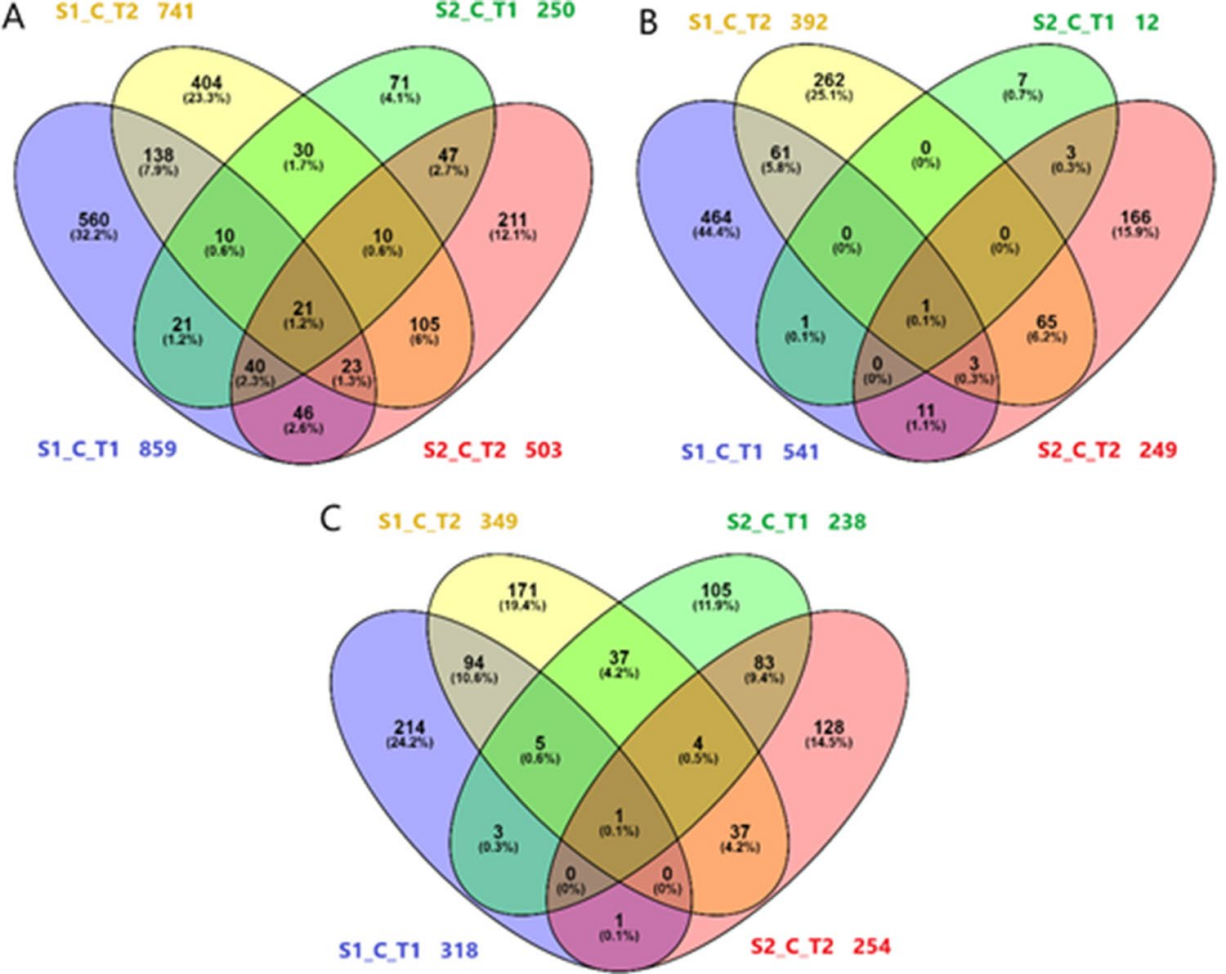
Venn diagrams displaying an overview of DEGs by T1/T2 compared with control in each stage of sampling, S1 and S2. (**A**) All DEGs, S1_C_T1: 859, S1_C_T2: 741, S2_C_T1: 250, S2_C_T2: 503; (**B**) upregulated DEGs, S1_C_T1: 541, S1_C_T2: 392, S2_C_T1: 12, S2_C_T2: 249; (**C**) downregulated DEGs, S1_C_T1: 318, S1_C_T2: 349, S2_C_T1: 238, S2_C_T2: 254.

Venn diagrams (Fig. 3) were used to depict the number of DEGs in response to T1 and T2 in each sampling stage. A total of 859 transcripts were deregulated by T1 in S1, of which 541 were upregulated and 318 downregulated. In contrast, 741 genes differed in expression level in T2 berries compared with controls, comprising 392 upregulated and 349 downregulated genes. At S2, 250 and 503 genes differed in expression level under T1 and T2, respectively compared to controls, comprising 12 and 249 upregulated and 238 and 254 downregulated genes, respectively (Fig. 3). The greatest number of DEGs was detected under T1 at S1, in agreement with the PCA results above. Of 859 DEGs, 560 were specific to T1 at S1, whereas only 71 of 250 DEGs changed under the same treatment (T1) in S2 (Fig. 3A).

Figure 3B,C shows that 464 and 214 up- and downregulated DEGs, respectively, were specific to the effect of T1 at S1, and only 7 and 105 up- and downregulated DEGs, respectively, were specific to the same treatment at S2.

Interestingly only 21 DEGs were commonly found to be deregulated among the four conditions (Fig. 3A, Table 2). Notably, Vitvi07g00381, annotated as an ATP binding protein was commonly induced (Fig. 3B, Table 2), and Vitvi03g00395, an *allene oxide synthase* (*AOS*) was commonly repressed (Fig. 3C, Table 2) in the four conditions.

**Table 2:** DEGs found to be deregulated in common among the four conditions: T1 compared to C at S1 (S1_C_T1), T2 compared to C at S1 (S1_C_T2), T1 compared to C at S2 (S2_C_T1) and T2 compared to C at S2 (S2_C_T2).

Cost_V3_ID	S1_C_T1	S1_C_T2	S2_C_T1	S2_C_T2	Functional annotation
Vitvi07g00078	5.195	2.109	− 2.022	− 2.135	Heat shock transcription factor A6B
Vitvi01g00408	3.818	2.519	− 1.083	− 1.473	Molecular chaperone DnaJ
Vitvi01g01708	6.128	2.807	− 2.683	− 2.487	BCL-2-associated athanogene 5
Vitvi02g00025	6.024	3.141	− 2.478	− 2.533	Heat shock protein 90-1
Vitvi02g01446	4.957	1.839	− 1.715	− 1.714	Heat shock protein MTSHP
Vitvi03g00395	− 1.843	− 2.154	− 3.071	− 1.972	AOS (allene oxide synthase)
Vitvi03g00568	− 1.018	− 4.087	− 2.439	1.969	Indole-3-acetate beta-glucosyltransferase
Vitvi04g00092	5.617	2.510	− 2.650	− 2.229	Heat shock transcription factor A6B
Vitvi05g00218	3.222	1.507	− 1.195	− 1.023	Mitochondrial substrate carrier family protein
Vitvi05g02185	2.767	1.274	− 1.573	− 1.330	No hit
Vitvi07g00381	1.453	2.172	1.077	2.415	ATP binding protein
Vitvi08g02189	3.029	2.054	− 1.203	− 1.611	Heat shock protein 70
Vitvi08g00689	3.459	1.323	− 1.079	− 1.094	DnaJ homolog. subfamily B. member 9
Vitvi09g02017	5.340	2.425	− 1.739	− 1.866	No hit
Vitvi09g00045	4.947	2.380	− 1.790	− 1.827	Small heat stress protein class CIII
Vitvi13g01107	2.940	1.292	− 1.571	− 1.563	Ripening regulated protein DDTFR8
Vitvi14g01946	− 1.762	− 2.266	− 2.467	1.273	Amino acid permease
Vitvi16g00681	4.627	2.339	− 1.586	− 2.086	Heat shock 22 kDa protein
Vitvi16g01103	4.605	1.432	− 1.551	− 1.368	Heat shock protein 90-1
Vitvi17g00695	3.538	1.416	− 1.366	− 1.707	Heat shock protein 101
Vitvi18g02720	3.579	− 2.697	− 2.079	1.419	Flavonoid 3-monooxygenase

When comparing DEGs between T2 and T1 at both stages it appears that the transcriptome of S1 showed a higher number of modulated genes than S2 (Supplementary Fig. S2). Compared to T1, T2 deregulated several genes related to Wounding, Desiccation stress response and ABA-mediated Signaling pathways, which was more pronounced in at S1 (Supplementary Table S2). This indicates a stronger modulation of pathways linked to plant innate immunity by coinfection (T2) in the early ripening berry.

### Identification of DEGs with similar mean expression patterns across all conditions

**Figure 4:**
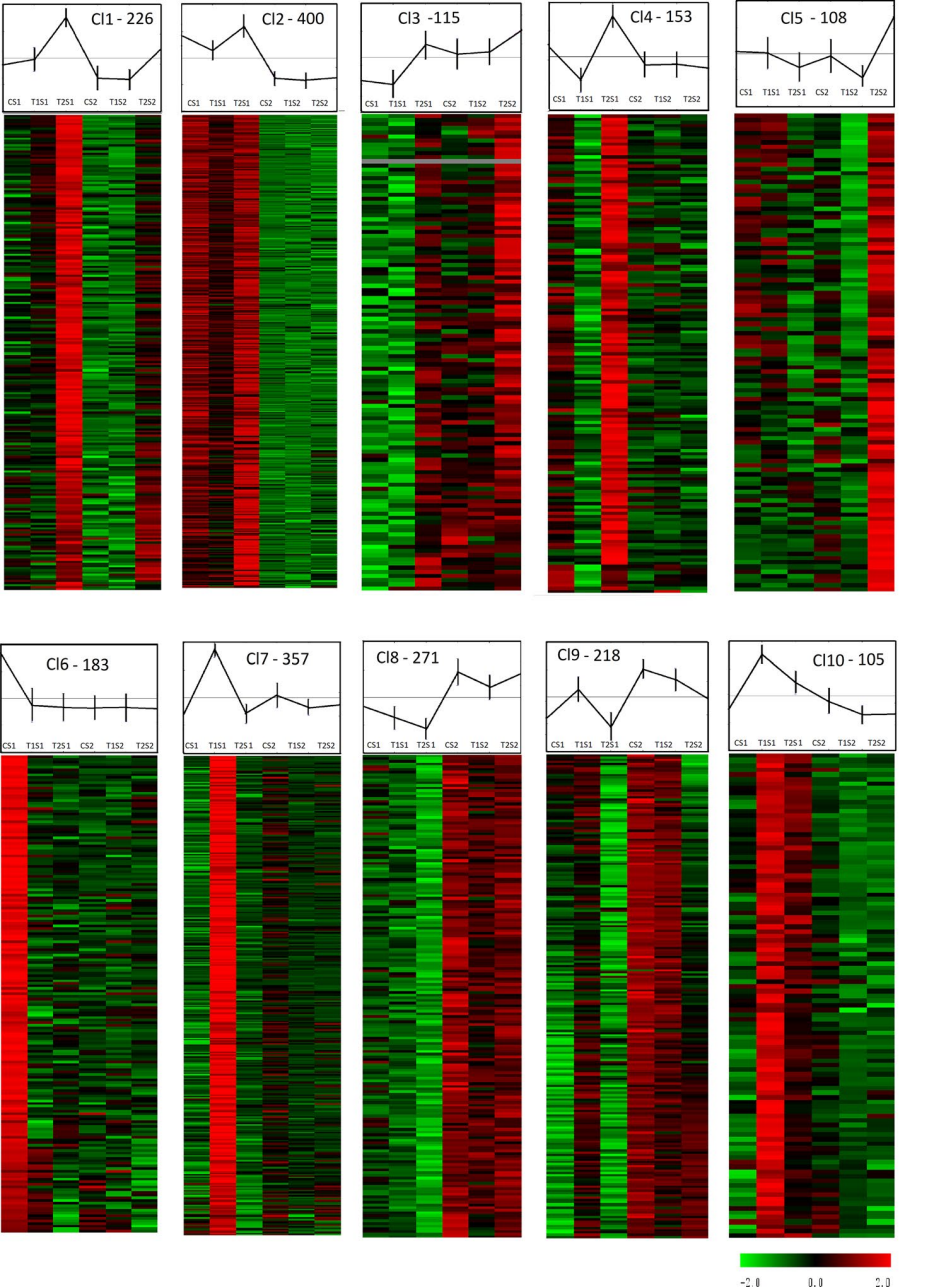
Expression profiles of T1 and T2 DEGs at stages S1 and S2. Clustering was performed by k-means on mean centered normalized expression log_2_ values. Hierarchical clustering was generated using the Multiple Experiment Viewer version 4.6.2 (https://mev.tm4.org/#/welcome).

To identify common patterns of gene regulation in a stage and treatment dependent manner, we allocated the 2,136 DEGs to 10 clusters through hierarchical k-means clustering (Fig. 4 and Supplementary Table S3) before analyzing the relative enrichment (p value ≤ 0.05) of functional categories (FC) within each cluster (Cl) (Supplementary Table S4).

Transcripts highly induced by T2 were mainly allocated to Cl1, Cl3 and Cl4. Genes within Cl1 and Cl3 were triggered by T2 in both developmental stages. Conversely, Cl4 was attributed to genes that showed T2 regulation only at S1 (Fig. 4).

Xyloglucan and pectin modification FCs prevailed in Cl1, whereas in Cl4, ABA-mediated signaling, abiotic stress response and heat shock mediated protein folding predominated (Supplementary Table S4). Expression profile of transcripts allocated to Cl9 were not so highly repressed by T2 compared to C at S1. Cl9 profile rather shows an inverse effect between T1 and T2 at S1. Enriched FCs in Cl9 were associated with starch catabolism (Vitvi04g00798, Vitvi17g01121), jasmonate-mediated signaling and phenylpropanoid biosynthesis specifically associated with stilbene biosynthesis and genes involved in lignin biosynthesis, such as *Ferulate-5-hydroxylase* (*F5H*: Vitvi04g01412), and also in lignan biosynthesis, such as *Pinoresinol-lariciresinol reductase* (*PLR*: Vitvi02g01001) (Supplementary Table S1).

Within Cl2 and Cl8, genes were predominantly regulated in a stage dependent manner rather than by viral infection and were dominated by gene categories related to flavonoid synthesis such as *flavanone 3-dioxygenase* (*F3H*: Vitvi02g00271, Vitvi09g00086) and a *Chalcone isomerase* (*CHI*: Vitvi14g01683) transcripts. Other genes within these clusters were associated with proton-dependent oligopeptide transporter and multidrug ATP-binding cassette transport (Supplementary Table S4).

## Discussion

Results from PCA (Fig. 2) and Venn diagrams (Fig. 3) indicate, that virus mediated gene expression was highest in the early ripening berry just after véraison. This high sensitivity of the early post-véraison berry to biotic and abiotic stresses has been highlighted in several previous studies^11,13,22^. However, Vega et al*.*^9^ obtained partly different results, in which the later ripening stage (E-L38) showed a higher number of DEGs than that at véraison (E-L35). In latter study, sampling did not account for berry heterogeneity which possibly explains results divergence.

Flavonoids, including flavonols, anthocyanidins and proanthocyanidins, are phenolic compounds that derive from the phenylpropanoid pathway which has been shown to be repressed in previous studies on berries from virus infected vines^9, 15^. In the present study, expression profiles differed considerably from previously reported results for the phenylpropanoid pathway and downstream branches. Only two isoenzymes of phenylalanine ammonia-lyase (*PAL1:* Vitvi08g01022 and *PAL2:* Vitvi13g00622), which catalyzes the first committed step of the phenylpropanoid pathway^23^, were slightly but not significantly repressed by T1 and T2 at both stages, S1 and S2 (Supplementary Table S5).

Further downstream of *PAL*, *cinnamate 4-hydroxylase* (*C4H*) catalyzes the conversion of cinnamate into p-coumarate, and then *4-coumarate CoA ligase* (*4CL*) ensures the formation of p-coumaroyl CoA. In the present study, *C4H* and *4CL* transcripts (Vitvi06g00803, Vitvi11g01257 and Vitvi17g00148) showed only a non-significant downregulation trend (Supplementary Table S5), and only one *C4H* transcript (Vitvi11g00924) was differentially repressed by T1 and T2 at S2 (Supplementary Table S1).

Concomitantly, further downstream *Chalcone synthase* transcripts (*CHS1*: Vitvi14g01448 and *CHS3*: Vitvi05g01044), encoding the first committed enzyme in flavonoid biosynthesis, which catalyzes the polyketide condensation reaction of p-coumaroyl-CoA and three acetate units from malonyl-CoA to yield chalcone^24^, were not significantly modulated by both GLD treatments. These results do not confirm those reported by Vega et al*.*^9^, who found that *CHS1* was upregulated in GLRaV-3 infected berries around véraison (E-L35–E-L36), whereas *CHS3* was downregulated at E-L35 and induced at E-L36.

**Figure 5:**
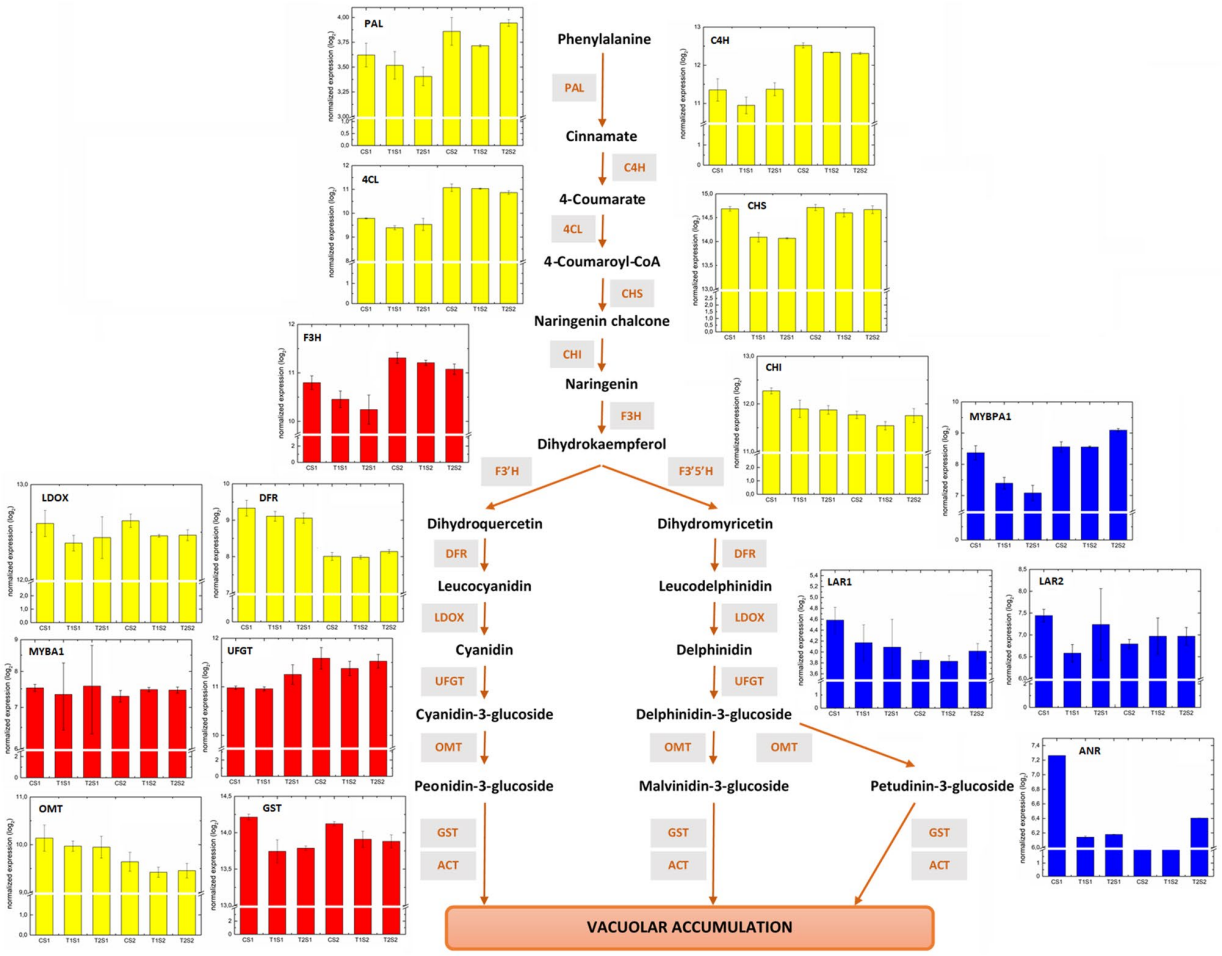
Overview of the effects of GLD infections on transcripts associated with anthocyanin synthesis at the two berry developmental stages. Relative gene expression data are means and standard deviation of normalized expression from the sequenced replicates.

Proanthocyanidins, or condensed tannins, consisting mainly of (+)-catechin, (−)-epicatechin, (−)-epigallocatechin and (−)-epicatechin 3-gallate, are regulated by the TF *MYBPA1* (Vitvi15g00938)^25^. This TF was found to be differentially repressed by T1, and more so by T2, but only at S1 (Fig. 5 and Supplementary Table S1). Concomitantly, the *MYBPA1* regulated transcripts *leucoanthocyanidin reductase* (*LAR1*: Vitvi01g00234; *LAR2*: Vitvi17g00371) and *anthocyanidin reductase* (*ANR*: Vitvi10g02185)^26^, which are responsible for producing the flavan-3-ol monomers required for the formation of proanthocyanidin polymers^27^, were found to be impaired by GLD at S1 (Fig. 5). This partial repression of proanthocyanidin synthesis by GLD at the first stage (S1) is consistent with results obtained by Vega et al*.*^9^.

Further downstream of proanthocyanidins, vacuolar anthocyanins are generated after cytosolic modification of anthocyanidin aglycones by glycosylation, methylation and acylation. In particular, glycosylation enhances the hydrophilicity and stability of anthocyanins and provides color stability^28^. A large family of *glycosyltransferases* (*GT*) ensure the O-glycosylation of anthocyanidins or anthocyanins in plants, among which the *UDP-glucose: flavonoid-3-O-glycosyltransferase* (*UFGT*) catalyzes the O-glycosylation of anthocyanidins at the C3 position in *V. vinifera* grapes^29^. Neither *VvUFGT* (Vitvi16g00156) nor *VvMybA1* (Vitvi02g01019; Fig. 5 and Supplementary Table S5), one of the main regulators of anthocyanin synthesis in grapes^30^, was affected by T1 and T2. Vega et al*.*^9^, have reported that *VvUFGT1* and *VvMYBA1* were strongly repressed by GLRaV-3 at ripening (E-L38), although *MYBPA1* was also repressed by the viral infection at the ripening stage (E-L38). This suggests that anthocyanin biosynthesis is not directly impaired by GLD infections and highlights that previously reported effects on anthocyanin biosynthesis caused by GLD infection^9^ are mainly due to a virus induced delay in the phenology of berries sampled at the same time point, which from a practical points of view is naturally of great importance and highlights the importance of both sampling strategies.

Grapevine berry quality is highly dependent on sugar accumulation, which starts at véraison, together with fruit softening and is followed by the onset of anthocyanin synthesis^16^. The accumulation of hexose sugars (glucose and fructose) in berries is well established to indicate the activity of sucrose-metabolizing enzymes, sucrose transporters and monosaccharide transporters^31^.

In the grapevine genome, a small gene family containing three members (*VvSUC11*/*SUT1*, *VvSUC12* and *VvSUC27*) encodes sucrose transporters in berries. The increase in *VvSUC11* and *VvSUC12* expression closely correlates with post-véraison sugar accumulation during ripening and suggests that *VvSUC11* and *VvSUC12* may have roles in the import of sucrose into ripening berry cells^31^.

In the study of Vega et al*.*^9^, transcript profiling showed that in GLRaV-3 infected vines, the accumulation of glucose and fructose decreased during ripening and that the expression of genes involved in sugar metabolism and transport was influenced by viral infections, particularly *VvHT1* and *VvMSA*, which were significantly downregulated during ripening.

**Figure 6:**
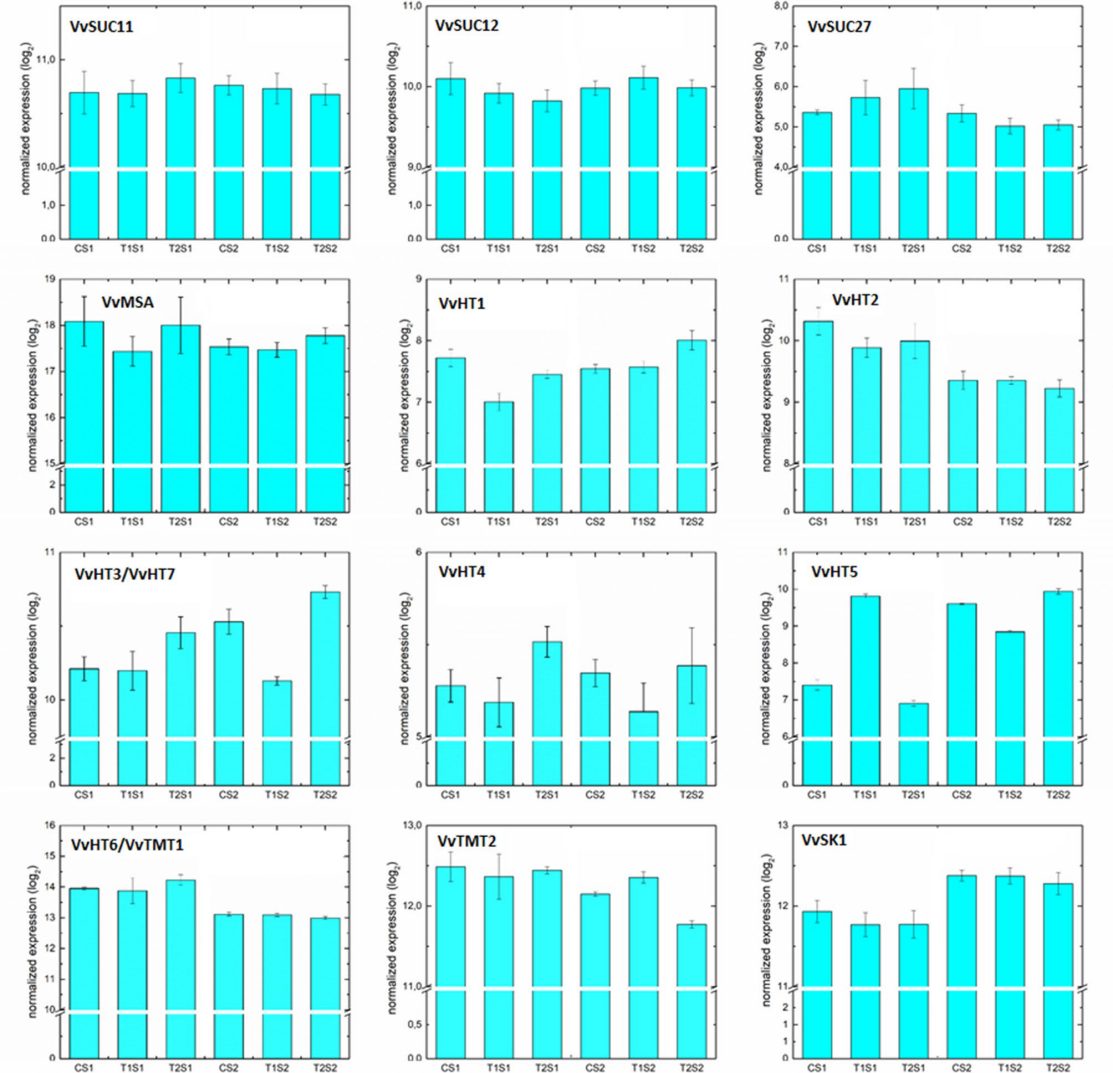
Effects of GLD treatments on principal transcripts associated with sugar metabolism during the two stages of development.

Here, no significant modulation of the three sucrose transporters *VvSUC11*/*SUT1* (Vitvi18g00584), *VvSUC12* (Vitvi01g00959) and *VvSUC27* (Vitvi18g01315), was observed in both stages. Moreover, none of the four *cytoplasmatic neutral invertases* (*nINV*: Vitvi03g00088, Vitvi05g00164, Vitvi13g00792 and Vitvi18g01682), which cleave sucrose into glucose and fructose, were significantly affected (Fig. 6 and Supplementary Table S6).

Six hexose transporters associated with glucose and fructose concentration have been identified in grape berries and named VvHT1–6. *VvHT1*, *VvHT2* and especially *VvHT3* are generally expressed more abundantly than the other *VvHT* genes in all stages of berry development^32^. In the present study, similarly to Vega et al*.*^9^, we found that *VvHT1* (Vitvi10g00358), the first transporter of this family identified in grapes^33^ and functionally characterized^34^, was significantly downregulated, but only by T1 at S1 (Supplementary Table S1). Moreover, its transcription factor *VvMSA* (Vitvi18g02973) was not significantly affected by GLD treatments (Fig. 6 and Supplementary Table S6).

Previously, Lecourieux et al*.*^35^ have shown that *VvSK1*, a protein kinase with sugar signaling function during berry development, positively affects sugar accumulation in grape cells and controls glucose transport through regulating four genes encoding the hexose transporters *VvHT3*, *VvHT4*, *VvHT5* and *VvHT6*. Here, *VvSK1* (Vitvi15g00840) was not significantly modulated by leafroll viral infections (Fig. 6 and Supplementary Table S6).

Except for the slight downregulation of *VvHT1*, the present results indicate only a very light repressive effect at the onset of véraison on sugar transport and metabolism, in agreement with the expression of anthocyanin biosynthesis genes, given that a tight positive correlation has been reported between sugar and anthocyanin concentrations in berries^36^. These expression patterns indicate that, as with anthocyanins, the decreased sugar accumulation in leafroll virus infected berries is caused by a virus induced ripening delay. Since sugar accumulation seemed to be slightly more impaired than anthocyanin synthesis (downregulation of *VvHT1*) virus infection might as well have caused a slight decoupling of sugar and anthocyanin accumulation as shown previously by temperature^37^.

In GLD infected berries several genes associated with innate plant defense mechanisms were upregulated in T1 and/or T2 infected berries at different stages. A *Leucine-rich repeat* family protein (*LRR*: Vitvi10g01162) was found to be upregulated by T2 compared to T1 at S1 (Supplementary Table S7), similarly to results reported by Vega et al*.*^9^ who showed that this gene was upregulated by GLRaV-3 in ripening berries. R proteins show direct interactions with Avr factors^38,39^, but most of them appeared to act indirectly via other intermediate host proteins. In the present study, several putative R genes were upregulated, mainly by T2 at S1 and S2 and downregulated by T1 at S1 (Supplementary Table S7), thus suggesting a more pronounced defense response triggered by T2 indicating that a coinfection with different viruses results in a stronger defense response of infected plants.

The role of jasmonic acid (JA) signaling in virus defense is controversial, we found several transcripts involved in the JA pathway to be affected by GLD (Supplementary Table S7). Interestingly, a *Lipoxygenase* protein (*LOX1*: Vitvi06g00155), the key enzyme in the JA pathway^40^, was strongly upregulated by T2 compared to T1 at S2, highlighting a stronger immune response by T2. Similarly, Vega et al*.*^9^ showed that *LOX2* protein expression increased in GLRaV-3 infected-berries at ripening (E-L38). Concomitantly, *Allene Oxide Synthase* (*AOS*: Vitvi18g00886), catalyzing the first committed step of JA biosynthesis by converting 13-HPOT to highly reactive allene oxide, which in the second committed step is converted to cis-12-oxo phytodienoic acid (OPDA) by *allene oxide cyclase* (*AOC*)^41^, was also induced by T2 at S1. Furthermore, other regulator genes affecting the JA pathway, such as *Enhanced Disease Susceptibility *1 protein (*EDS1*: Vitvi17g01523) was upregulated in T2 infected plants at S2, in a process putatively mediated by JA signaling. *EDS1* is considered a master redox sensitive transcriptional regulator of the salicylic acid response and a mediator of crosstalk between salicylic acid and JA^42^. It plays a significant role in the establishment of systemic acquired resistance and induced systemic resistance^43^ and affects the regulation of pathogenesis related gene expression^42,44^. Consequently, other pathogenesis related proteins (PR-1) transcripts (Vitvi18g02836, Vitvi18g01248) were induced by T2, highlighting the triggering of innate immune response.

Expression pattern of WRKY TFs (Vitvi07g01847, Vitvi08g00868, Vitvi12g01676, Vitvi13g01916) point as well in the direction of an enhanced activation of plant innate immunity by leafroll infection (Supplementary Table S8). Two transcripts coding for plant-specific transcription factor *WRKY70* (Vitvi08g00868 and Vitvi13g01916) were upregulated in T2 berries. *WRKY70* is a common component in salicylic acid (SA)- and jasmonic acid (JA)-mediated signal pathways and identified to be involved in the crosstalk between SA and JA-dependent defense signaling in plants^45^. Other studies indicate that *WRKY70* has a pivotal role in determining the balance between SA-dependent and JA-dependent defense pathways^46^. Together these expression patterns indicated that GLD infections trigger different autodefense mechanisms in grapevine berries and highlight that the response is significantly more pronounced when vines were infected with both virus particles (GLRaV-1 and GLRaV-3).

Genes within Oil body organization and biogenesis were induced by T1 in S1 and repressed by T2 at the same stage (Table 3 and Supplementary Table S8). 8 *xyloglucan endotransglycosylase*/*hydrolase* (*XTH*) transcripts were found down regulated in T2 samples (Supplementary Table S8). 3 among them (Vitvi05g02111, Vitvi11g01675 and Vitvi11g01677) were as well repressed within T1 samples. These expression pattern could point to a virus induced delay in berry growth, which is interestingly since it is even observable on a molecular level when berry samples were reconstituted according to their sugar and acid content. However, this could as well suggest that sample reconstitution was not homogenously enough as seen by the lower average sugar concentration of T1 and T2 samples, which might explain a part of the observed differences.

**Table 3:** Enrichment in functional categories, illustrated as relative odds ratio (log e) for upregulated and downregulated DEGs relative to the whole grapevine genome within T1 samples compared to C, within T2 samples compared to C and within T2 samples compared to T1, respectively, at both stages S1 and S2, (S1_C_T1, S2_C_T1, S1_C_T2, S2_C_T2, S1_C_T2 and S2_C_T2).

Functional categories	S1_C_T1	S1_C_T2	S2_C_T1	S2_C_T2	S1_T1_T2	S2_T1_T2
**Upregulated**						
HSP-mediated protein folding	3.16	2.03	X	X	X	X
Storage proteins	1.82	X	X	X	X	X
Antenna proteins	X	3.62	X	X	3.58	X
Photosystem I	X	2.75	X	X	2.91	X
Photosystem II	X	2.58	X	X	2.88	X
tRNA processing	X	3.07	X	X	X	X
Phenylalanine biosynthesis	X	X	X	2.11	X	1.84
Tyrosine catabolism	X	X	X	X	2.71	X
Starch catabolism inhibitor	X	X	X	X	X	4.17
Flavonoid biosynthesis	X	X	X	X	1.72	X
Desiccation stress response	2.9	X	X	X	X	X
Biotic stress response	X	X	X	X	X	1.76
bZIP family transcription factor	2.09	X	X	X	X	X
NAC family transcription factor	X	2.04	X	2.64	X	2.09
mRNA cleavage involved in gene silencing by miRNA	X	2.9	X	X	X	X
Pentatricopeptide domain family	− 1.80E + 304	X	X	X	X	X
Oil body organization and biogenesis	3.23	X	X	X	X	X
Autotransporter	X	X	X	X	2.11	X
**Downregulated**						
HSP-mediated protein folding	X	X	2.86	3.13	2.87	X
Protease inhibition	X	X	3.23	X	X	X
Storage proteins	X	X	X	X	1.83	X
Starch catabolism inhibitor	X	X	4.62	X	X	X
Tyrosine catabolism	X	X	3.17	X	X	X
Phenylpropanoid biosynthesis	X	1.92	2.76	X	X	X
Wounding	X	3.73	X	4.17	3.33	4.76
Desiccation stress response	X	X	X	X	2.93	X
Biotic stress response	X	X	2.3	1.74	1.24	X
Xyloglucan modification	X	2.88	X	X	X	X
Cell wall structural protein	X	X	X	X	X	2.83
Oil body organization and biogenesis	X	3.67	X	X	3.68	X
Protein kinase	X	X	X	X	-1.03	X
ABA-mediated signaling pathway	X	X	X	X	1.48	X
Pentatricopeptide domain family	X	X	X	X	− 1.80E + 304	X

Only categories with adjusted p-values lower than the 0.05 threshold are shown.

Interestingly in the present study Heat shock response showed opposed expression by GLD at S1 and S2 (Table 3 and Supplementary Table S8). At S1, heat shock related FCs including protein folding, chaperon mediated protein folding and HSP-mediated protein folding, were upregulated by T2. This enhanced heat shock response is particularly marked in Cl4 but might be mediated more by external factors than by virus infection and is putatively owed to the fact that berries from T2S1, although selected according to their biochemical composition, were phenologically the least advanced in comparison to T1S1 and CS1. It has been shown that heat shock response is more pronounced in early berry developmental stages close to véraison^20^ and might have been triggered by high mid-day temperatures in the vineyard during sampling.

## Conclusion

The present work is, to our knowledge, the first comprehensive molecular study conducted on GLD infected grapevine berries, which compares different viral agents in one experimental plot, thus under very homogeneous environmental conditions. The reconstitution of single berries according to their biochemical characteristics enabled us to mitigate the high intra-cluster heterogeneity and reduced GLD induced phenological shifts. The present data shows that GLD does almost not directly repress anthocyanin and sugar-transport related transcripts, which, from a grower perspective, does not curtail the negative effects of GLD on berry and wine quality, but provides new molecular insights from a fundamental physiological point of view.

Our study highlights the stress responsiveness of the early ripening berry just going through véraison and confirms findings from previous biotic and abiotic stress studies but has never previously been shown for viral infections. Interestingly, quantitative transcriptomic changes induced by viral infection were highest after GLRaV1 infection and not by a co-infection with two virus agents. However, detailed DEG analysis revealed that key transcripts mainly related to autodefense mechanisms regulated by JA-ET antagonism were most pronounced after co-infection with GLRaV-1 + GLRaV-3 (T2). This upregulation of plant innate immunity provides valuable information for resistance breeding programs and research aiming to trigger plant immune response by elicitors. Thus far, no major sources of virus resistances have been found in *V. vinifera* consequently future strategies could focuses on plant innate immunity by building up on results from our and other transcriptomic observations.

Nevertheless, it needs to be considered that our study was limited to one, relatively sensitive cultivar whereas it is well known that there are considerable genotypic differences in the susceptibility to virus infection, thus molecular results may vary in different cultivars. Comparing transcriptomic changes induced by virus infection reported here with more tolerant cultivars could elucidate potential resistance mechanism and pave the way for the breeding of more tolerant cultivars.

In the present study we did not analyze how virus modulated gene expression varies between different berry tissues during berry development which is certainly an interesting point to take into account in future studies since tissue specific gene expression does change along berry development in healthy vines^47^. Furthermore, it can be expected that molecular results are modulated by environmental conditions during the growing season as it is empirically known that virus symptoms change between vintages.

A further point to be considered is the heterogeneous distribution of virus particles between individual berries^7^, which could not be addressed within the scope of the present study. Although, we circumvented the problem of berry heterogeneity, however we cannot conclude about the different virus loads of individual berries, which was certainly different and biased our molecular results. It would thus be very interesting to address this question by analyzing berries individually and eventually perform single cell transcriptomics of individual berries from GLD infected vines.

Our study together with previous studies emphasizes the importance of the use of healthy, virus tested plant material when planting new vinyards to minimize virus propagation.

## Methods

### Plant material

Treatments consisted of 3 × 10 *Pinot noir* vines of clone RAC 68 selected from Agroscope Switzerland. Vine cuttings were obtained from certified vineyards from the Agroscope clonal selection program (no voucher specimen of this material has been deposited in a publicly available herbarium) and were infected by grafting (on 5BB rootstock) with GLRaV-1 (Treatment 1: T1), co-infected with GLRaV-1&3 (Treatment 2: T2) or uninfected (control: C). Grapevine plants were planted in the Agroscope grapevine virus collection (Switzerland; coordinates: 46° 23′ 50.3″ N 6° 13′ 57.9″ E), row orientating was north–south^48^. Samples were collected at two different ripening stages be during 1 h between 1 and 2 pm. The first stage (S1) was sampled at véraison (14.08.2017). As a convention, véraison stage was considered to be achieved when approximately 50% of berries were colored. Approximately 100 berries from five vines per treatment were randomly chosen, only berries that were colored (visually assessed) were chosen. The second sampling (S2) was performed 15 days later (29.08.2017) at mid-ripening on the five other vines not sampled during S1. To avoid splitting during freezing, the collected berries were individually covered in aluminum foil, immediately frozen in liquid nitrogen and subsequently stored at – 80 °C until further processing.

All berries were individually crushed, the seeds were removed, and 100 mg aliquots were prepared for organic acid and sugar analysis. The results were used to distinguish the developmental stage of each individual berry and to produce homogeneous batches (according to their sugar and organic acid content) for RNA extraction and subsequent RNA-seq analysis. No permissions were required to collect the plants.

To assess vine physiology leaf photosynthesis was evaluated by gas exchange measurements on ten well exposed adult leaves per treatment at the 10th of august 2018 during mid-day using a Ciras 3 Portable Photosynthesis System (PP Systems, USA). For the control of environmental parameters, gas exchange photosynthetically active radiation (PAR) inside the leaf cuvette was adjusted to 1,500 mmol m^−2^ s^−1^, temperature to 30 °C, relative humidity to 80% and a CO_2_ concentration to 400 ppm. Leaf chlorophyll fluorescence was as well performed the same day after gas exchange measurements with the Ciras 3 equipped with a CFM-3 Chlorophyll Fluorescence Module. Minimal level of fluorescence F0 and maximal possible value for fluorescence (F_m_) were measured and variable fluorescence F_v_ = F_m_ − F0 and quantum yield (F_v_/F_m_) were calculated. Leaves were dark adapted for 20 min before measurements. Yield was assessed in 2017 at harvest of all vines. N-test measurements were performed the 8th of august on 30 adult leaves per treatment.

The viral infections were monitored using commercially available ELISA kits (Bioreba AG, Switzerland) according to the manufacturer’s instructions. Beside GLRaV-1 and -3, other viruses regulated in the Swiss certification scheme (Grapevine leafroll associated virus 2 and 4, Grapevine fanleaf virus, Arabic mosaic virus, Raspberry ringspot virus, Tomato blackring virus and Strawberry latent ringspot virus) were screened by ELISA and were found to be non-present in our assay. Furthermore, PCR-based diagnostic assays were used to confirm viral infection on every single vine in our experiment. RNA extracts were performed from dormant canes during winter using the CTAB method^49^. Samples were screened for GLRaV-1 by end point RT-PCR using the primers LQV1-H47 and LEV1-C447, as published by Osman and Rowhani^50^. GLRaV-3 samples were screened using the RT-qPCR assay published by Diaz-Lara et al.^51^ according to authors ‘specifications. Amplified DNA was separated on 1.5% agarose gels and stained with ethidium bromide.

### Organic acid and sugar chromatography analysis

Organic acids and sugar were analyzed with HPLC on a 1,260 Infinity Agilent HPLC system. Approximately 100 mg of N_2_-ground sample powder was diluted in 1 mL of deionized water and homogenized. Then 500 µL of sample solution was added to 4.5 mL of 0.65 mM sulfuric acid solution (H_2_So_4_), pre-treated by solid phase extraction with Waters Oasis HLB, 6 cm^3^ (200 mg) cartridges (Waters Corporation, Milford, MA, USA) and subsequently filtered through 0.2 µm nylon filters (Millipore, Burlington, MA, USA). A volume of 20 μL per sample was injected onto an Aminex HPX-87H column 300 × 7.8 mm (Bio-Rad Laboratories, Hercules, CA, USA) and eluted under isocratic conditions at 80 °C with 0.65 mM H_2_SO_4_ solution mobile phase at a 0.5 mL min^−1^ flow rate. Eluting organic acids were detected with UV absorbance (210 nm), and the refractive index was measured with a Waters 2487 dual absorbance detector (Waters Corporation, Milford, MA, USA). A Kontron 475 refractive index detector (Kontron Instruments, Rossdorf, Germany) was used to determine fructose and glucose concentrations. Concentrations were calculated according to Eyéghé-Bickong et al*.*^52^.

### RNA extraction and RNA-seq analysis

A total of 18 ground samples were reconstituted as three biological samples from the three treatment cases and the two stages of development. Total RNA extraction was performed as described by Rienth et al*.*^53^. A Quawell Q9000 spectrophotometer (Labgene Scientific SA) was used to evaluate RNA concentration and purity by measuring the absorbance at 260 and 280 nm. Only samples with an A260/A280 ratio greater than 1.8 were retained for further analyses. RNA integrity was assessed with an Agilent 2100 Bioanalyzer (Agilent Technologies). All samples had RNA integrity numbers of 8 or higher. Three replicates per treatment were analyzed.

### Library preparation for transcriptome sequencing

TruSeq stranded mRNA-seq (Illumina, San Diego, CA, USA) was used for library preparation according to the manufacturer’s instructions. Both RNA samples and final libraries were quantified with a Qubit 2.0 fluorometer (Invitrogen) and quality tested with an Agilent 2100 Bioanalyzer RNA Nano assay (Agilent Technologies, Santa Clara, CA, USA). Libraries were then sequenced with 75 bp single-end mode with a NextSeq500 apparatus (Illumina).

### Gene expression analysis

Gene counts were computed by summing counts of different transcripts of the same gene. Then gene read counts were transformed by the regularized logarithm (*rlog*) by using R version 3.4.4^54^ (R Development Core Team), and PCA was performed on the gene expression log transformed values. This transformation removed the dependence of the variance on the mean and normalized count data with respect to library size.

DEGs were identified with the R package DESeq^55^. Pairwise comparison was performed between uninfected (C) and virus-infected (GLRaV-1 and GLRaV-1&3) conditions at the two sampling stages separately. Transcripts were considered significantly modulated or differentially expressed according to the following criteria: absolute fold change > 2 (log_2_ fold change < − 1; > + 1) and FDR-adjusted p-value < 0.05. Gene annotation was derived from Grimplet et al*.*^56^ and new gene identified were derived from Canaguier et al.^57^. Venny 2.1 tool^58^ was used to draw Venn diagrams to compare the lists of DEGs identified under each treatment and stage. To understand the underlying biological processes, a k-means clustering analysis was performed with the Multiple Experiment Viewer version 4.6.2, on normalized mean centered logs of DEGs (Supplementary Table S9). Functional categories were derived from Grimplet et al*.*^56^. Subsequently, to identify significant enrichment in functional categories, we performed Fisher’s exact test to compare the gene list with non-redundant transcripts from the grapevine genome with the FatiGO analysis tool^59^. Multiple testing was adjusted for with the BH approach, and a BH adjusted p-value < 0.05 was considered to indicate enrichment.

## Supplementary information

Supplementary Figure S1.

Supplementary Figure S2.

Supplementary Information.

Supplementary Table S1.

Supplementary Table S2.

Supplementary Table S3.

Supplementary Table S4.

Supplementary Table S5.

Supplementary Table S6.

Supplementary Table S7.

Supplementary Table S8.

Supplementary Table S9.

## Data Availability

The raw sequence reads of the 18 samples have been uploaded to NCBI and are publicly available as fastq files in the Sequence Read Archive (SRA) database of the National Center for Biotechnology Information (NCBI) under the reference PRJNA594635 and the following link https://www.ncbi.nlm.nih.gov/sra/PRJNA594635 under the accession codes SAMN13525331, SAMN13525332, SAMN13525333, SAMN13525334, SAMN13525335, SAMN13525336, SAMN13525337, SAMN13525338, SAMN13525339, SAMN13525340, SAMN13525341, SAMN13525342, SAMN13525343, SAMN13525344, SAMN13525345, SAMN13525346, SAMN13525347 and SAMN13525348.
