# Supplementary figures and images for: Single berry reconstitution prior to RNA-sequencing reveals novel insights into transcriptomic remodeling by leafroll virus infections in grapevines
Source: Sci Rep. 2020 Jul 31;10:12905. doi: 10.1038/s41598-020-69779-1 (PMC7395792; doi:10.1038/s41598-020-69779-1)

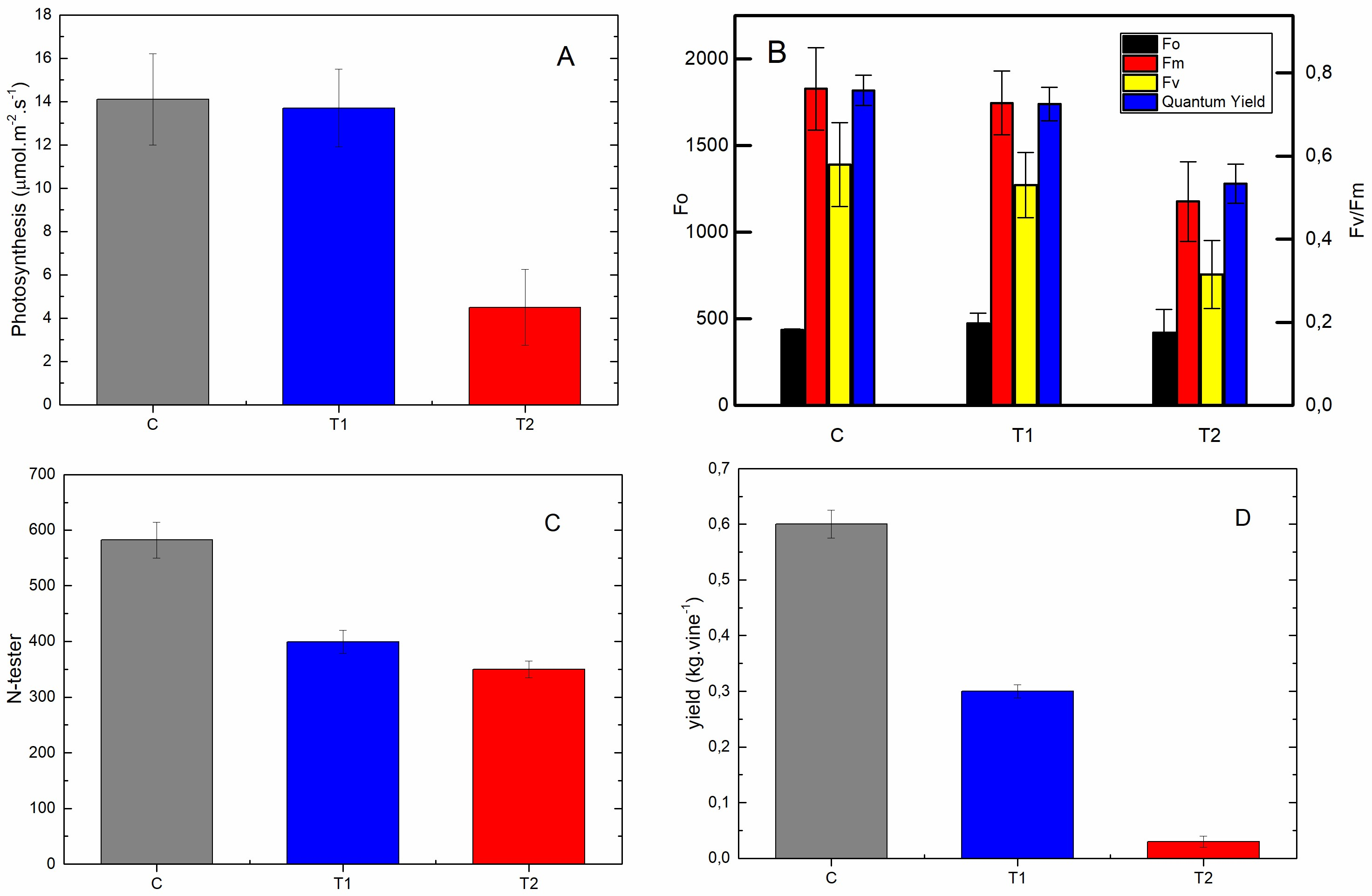

Supplement: Supplementary file 1 — Supplementary Figure S1. [file 41598_2020_69779_MOESM1_ESM.tiff]

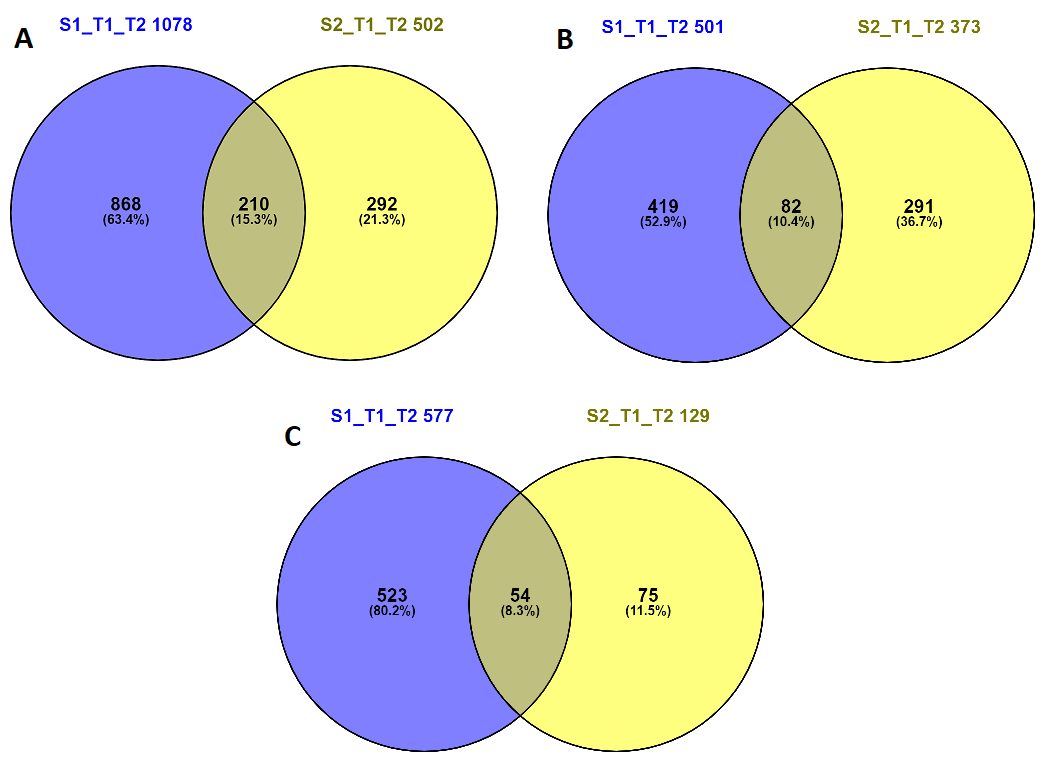

Supplement: Supplementary file 2 — Supplementary Figure S2. [file 41598_2020_69779_MOESM2_ESM.tiff]
