# Supplementary material for: Single berry reconstitution prior to RNA-sequencing reveals novel insights into transcriptomic remodeling by leafroll virus infections in grapevines
Source: Sci Rep. 2020 Jul 31;10:12905. doi: 10.1038/s41598-020-69779-1 (PMC7395792; doi:10.1038/s41598-020-69779-1)
Supplement: Supplementary file 3 — Supplementary Information. [file 41598_2020_69779_MOESM3_ESM.docx]

**Supplementary Information Title page**

**Single berry reconstitution prior to RNA-sequencing reveals novel insights into transcriptomic remodeling by leafroll virus infections in grapevines**

Sana Ghaffari^1, 2^, Jean Sébastien Reynard^3^ & Markus Rienth^1*^

^1^ CHANGINS – Changins, HES-SO University of Applied Sciences and Arts Western Switzerland, College for Viticulture and Enology, Nyon, Switzerland

^2^ Higher Institute of Applied Biology of Medenine, Medenine, Tunisia

^3^ Virology-Phytoplasmology Laboratory, Agroscope, Nyon, Switzerland

^*^email: [markus.rienth@changins.ch](mailto:markus.rienth@changins.ch)

**Supplementary Information legends**

**Supplementary Fig. S1.** A: Leaf photosynthesis, B: Chlorophyll fluorescence: F_0_ minimal level of fluorescence, F_m_ maximal possible value for fluorescence, F_v_ variable fluorescence. C: yield in kg.vine^-1^, D: N-tester.

**Supplementary Fig. S2.** Venn diagrams displaying an overview of T2 versus T1 DEGs in each stage of sampling, S1 and S2. A: All DEGs, S1_T1_T2: 1078, S2_T1_T2: 502; B: Upregulated genes, S1_T1_T2: 501, S2_T1_T2: 373; C: Downregulated genes, S1_T1_T2: 577, S2_T1_T2: 129.

**Supplementary Table S1.** Differentially expressed genes (DEGs) (lfc > 1, padj < 0.05) between C, T1 and T2, at stages S1 and S1.

**Supplementary Table S2.** Enriched functional categories from genes groups of Supplementary Fig. S2, illustrated as relative odds ratio (log e). Only categories with adjusted p-values lower than the 0.05 threshold are presented.

**Supplementary Table S3.** DEGs allocated to clusters.

**Supplementary Table S4.** Enriched functional categories within each cluster.

**Supplementary Table S5.** Anthocyanin related genes.

**Supplementary Table S6.** Sugar metabolism related genes**.**

**Supplementary Table S7.** Defense related genes in response to GLD.

**Supplementary Table S8.** DEGs related to Cellular component organization and biogenesis, HSPs and transcription factors.

**Supplementary Table S9.** DEG mean centered normalized expression log_2_ values.
